# Low Frequency Extension Filter and ActiGraph-Calculated Sleep Intervals in Older Adults

**DOI:** 10.1093/geroni/igaa057.1381

**Published:** 2020-12-16

**Authors:** Hilary Hicks, Alex Laffer, Genna Losinski, Amber Watts

**Affiliations:** The University of Kansas, Lawrence, Kansas, United States

## Abstract

Actigraphy has become a popular, non-invasive means of continuously monitoring physical activity and sleep. One optional setting, the low frequency extension (LFE) filter, reduces the movement threshold to capture low acceleration activity that is common in older adults. This filter significantly alters physical activity outcomes (e.g., step counts), but it is unclear if this has implications for sleep interval calculations that rely upon accurate differentiation between physical activity and sleep. We investigated the effects of the LFE filter on wrist-worn sleep estimates in older adults. Participants were 9 older adults who wore the ActiGraph GT9X on their non-dominant wrist for 7 days in a free-living environment. Raw data was processed with and without the LFE filter enabled, and sleep intervals were calculated by a proprietary ActiGraph algorithm. Paired samples t-tests demonstrated that the LFE filter generated significantly later bedtimes, fewer minutes spent in bed, shorter sleep duration, and fewer awakenings during the night compared to when the filter was disabled (all p < .043). Use of the LFE filter did not lead to differences in arise time, sleep latency, efficiency, or wake after sleep onset (all p > .052). While the LFE filter was designed to improve accuracy of physical activity estimates in more sedentary populations, these findings suggest that the LFE filter also has the potential to impact sleep estimates of older adults. Researchers using ActiGraph-calculated sleep would benefit from careful consideration of this software-dependent impact.

